# Cardiovascular Outcomes Among Patients with Acute Coronary Syndromes and Diabetes: Results from ACS QUIK Trial in India

**DOI:** 10.5334/gh.1290

**Published:** 2024-04-24

**Authors:** Abdulhamied Alfaddagh, Haitham Khraishah, Giulio R. Romeo, Mohamad B. Kassab, Zeb McMillan, Nisha Chandra-Strobos, Roger Blumenthal, Mazen Albaghdadi

**Affiliations:** 1 Ciccarone Center for the Prevention of Cardiovascular Disease, Division of Cardiology, Johns Hopkins University School of Medicine, Baltimore, MD, US; 2 Department of Medicine, Beth Israel Deaconess Medical Center, Harvard Medical School, Boston, MA, US; 3 Joslin Diabetes Center, Harvard Medical School, Boston, MA, US; 4 Cardiovascular research center, Massachusetts General Hospital, Boston, MA, US; 5 Department of Anesthesiology, Division of Critical Care, UC San Diego, San Diego, CA, USA; 6 Division of Cardiology, Johns Hopkins University School of Medicine, Baltimore, MD, US; 7 Division of Cardiology, Massachusetts General Hospital, Harvard Medical School, Boston, MA, US

**Keywords:** Diabetes, myocardial infarction, cardiovascular outcomes, India, lower-middle income country

## Abstract

**Background:**

Despite cardiovascular disease being the leading cause of death in India, limited data exist regarding the factors associated with outcomes in patients with diabetes who suffer acute myocardial infarction (AMI).

**Methods:**

We examined 21,374 patients with AMI enrolled in the ACS QUIK trial. We compared in-hospital and 30-day major adverse cardiac events including death, re-infarction, stroke, or major bleeding in those with and without diabetes. The associations between diabetes and cardiac outcomes were adjusted for presentation and in-hospital management using logistic regression.

**Results:**

Mean ± SD age was 60.1 ± 12.0 years, 24.3% were females, and 44.4% had diabetes. Those with diabetes were more likely to be older, female, hypertensive, and have higher Killip class but less likely to present with STEMI. Patients with diabetes had longer symptoms onset-to-arrival (median 225 vs 290 min; P < 0.001) and, in case of STEMI, longer door-to-balloon times (median, 75 vs 91 min; P < 0.001). Diabetes was independently associated with higher in-hospital death (adjusted odds ratio [aOR], 1.46; 95% CI, 1.12–1.89), in-hospital reinfarction (aOR, 1.52; 95% CI, 1.15–2.02), 30-day MACE (aOR, 1.33; 95% CI, 1.14–1.55) and 30-day death (aOR, 1.40; 95%CI, 1.16–1.69) but not 30-day stroke or 30-day major bleeding.

**Conclusion:**

Among patients presenting with AMI in Kerala, India, a considerable proportion has diabetes and are at increased risk for in-hospital and 30-day adverse cardiovascular outcomes. Increased awareness of the increased cardiovascular risk and attention to the implementation of established cardiovascular therapies are indicated for patients with diabetes in lower-middle-income countries who develop AMI.

**Clinical Trial registration:**

ClinicalTrials.gov Unique identifier: NCT02256658.

## Introduction

Despite effective public health efforts in decreasing the incidence of ischemic heart disease (IHD) since the 1960s in high-income countries (HICs), IHD continues to exert a significant toll in low- and middle-income countries (LMIC) [1]. Approximately 80% of cardiovascular (CV) deaths occur in LMICs, with 40% of deaths labeled as premature [2]. Literature relevant to South Asian populations and CV health has demonstrated a higher incidence of IHD that affects individuals at a younger age who experience worse outcomes compared to other ethnic groups [1,3,4]. This is likely related to the increased burden of traditional risk factors among individuals of South Asian heritage [5]. Diabetes mellitus is a potent risk factor for cardiovascular disease (CVD) and increases the risk of IHD by 2–3 fold [6]. The burden of diabetes has steadily increased over the past 25 years in India and worldwide, with India contributing a major part of the global burden [7]. Between 1990 to 2016, the adult population with diabetes has increased from 26.0 million to 65.0 million, with highest prevalence in Kerala [7]. Using data from the Acute Coronary Syndrome Quality Improvement in Kerala (ACS QUIK) randomized clinical trial, we performed a sub-study to investigate the effect of diabetes status on the clinical presentation and cardiovascular outcomes after acute myocardial infarction (AMI).

## Methods

### Study design and population

We examined patients presenting with AMI enrolled in the ACS-QUIK trial. The full details of the trial design were published elsewhere [8]. In brief, ACS QUIK was a large, multicenter, cluster-randomized, stepped-wedged pragmatic clinical trial that examined the role of a quality improvement tool kit on CV outcomes. A total of 63 hospitals located in Kerala, India, were randomized with a total of 21,374 patients enrolled between November 10, 2014, and November 9, 2016. Eligible patients had to be ≥18 years of age and present to one of the participating hospitals with either non-ST-segment myocardial infarction or ST-segment elevation myocardial infarction (STEMI) as defined by the Third Universal Definition of Myocardial infarction [9]. ACS QUIK was approved by the ethics boards of local, national, and international bodies and was approved by the Indian Health Ministry Screening Committee. All patients provided written informed consent prior to their participation.

### In-hospital data collection

Patient-level in-hospital data were collected based on the 2013 ACCF/AHA key data elements and definitions for measuring the clinical management and outcomes of patients with acute coronary syndromes and coronary artery disease [10]. In-hospital data included the patient’s medical history, demographics, previous and concomitant medications, presenting vital signs and physical exam findings, and initial blood tests. Patients were recorded as having diabetes if they had a history of diabetes diagnosed and/or treated by a healthcare provider.

Therapies given on admission were recorded. This included the administration of aspirin, adjuvant antiplatelet therapy, beta-blockers, adjuvant anti-coagulation therapy, and primary reperfusion therapy. Primary reperfusion therapy for eligible patients with ST-segment elevation myocardial infarction included primary percutaneous coronary intervention or primary fibrinolytic therapy. Subsequent diagnostic angiography or PCI during admission were also recorded. Treatments received during hospitalization and discharge were also recorded.

### Study outcomes

The primary outcome of this analysis was a composite endpoint of 30-day MACE including death, re-infarction, stroke, or major bleeding. Reinfarction was defined according to the Third Definition of Myocardial Infarction [9]. Major bleeding was defined using the Global Utilization of Streptokinase and Tissue Plasminogen Activator for Occluded Coronary Arteries (GUSTO) criteria [11]. We also analyzed 30-day death, 30-day CVD death, and in-hospital adverse events including in-hospital death, re-infarction, major bleeding, stroke, heart failure, cardiogenic shock, and cardiac arrest.

### Statistical analysis

Categorical variables were summarized by their proportions and continuous variables were summarized by their means (SD) or medians (IQR) where appropriate. Proportions were compared between groups using a Chi^2^ test. Normally distributed continuous variables were compared using Student t-test and skewed variables were compared using Mann-Whitney-U test.

Logistic regression was used to estimate the odds ratios and 95% confidence intervals for the associations between diabetes status and adverse outcomes. Standard errors of the estimated associations were adjusting for clustering by center. We presented unadjusted associations and performed hierarchical multivariable modeling whereby model 1 adjusted for clustering by center. Model 2 adjusted for clustering by center plus clinically important baseline factors including age, sex, smoking status, presence of STEMI, systolic blood pressure, heart rate, Killip class, initial creatinine, and troponin values. Model 3 adjusted for factors included in model 2 plus in-hospital treatments including administration of aspirin, anticoagulation, and PCI during admission. Model 3 was also adjusted for the quality improvement treatment arm that a given healthcare center was randomized to during the ACS-QUIK trial. Multiplicative interaction terms were calculated to test whether the association between diabetes and adverse outcomes differed by age, sex, history of hypertension, smoking status, presence of STEMI, receiving in-hospital aspirin or PCI during admission, and discharge medications. A 2-sided P-value < 0.05 was considered statistically significant. All analyses were performed using Stata Statistical Software version 16.1 (StataCorp LLC, TX).

## Results

### Clinical characteristics on presentation

Among the 21,374 participants included in this analysis, the mean ± SD age was 60.1 ± 12.0 years and 24.3% were females. A total of 9,484 (44.4%) participants had diabetes. A total of 13,689 (64.0%) presented with a STEMI, and 13.6% presented with a Killip class of 2 or greater. Table 1 shows the demographic and clinical characteristics at baseline in the total population and stratified by diabetes status. At baseline, patients with diabetes were more likely to be older, female, non-smokers, and have a history of peripheral arterial disease. Patients with diabetes were more likely to have longer symptom-to-hospital arrival times (median time, 290 vs 225 minutes; P < 0.001), have elevated Killip class on presentation (proportion with Killip class ≥2, 20.1% vs. 11.1%; P < 0.001), but less likely to be diagnosed with a STEMI (57.7% vs. 69.1%; P < 0.001). The baseline demographic and clinical characteristics in patients with diabetes with and without STEMI are shown in supplemental Table 1.

**Table 1:** Baseline demographic and clinical characteristics in the total group and by diabetes status.


CHARACTERISTICS	TOTAL (n = 21374)	NO DIABETES (n = 11890)	DIABETES (n = 9484)	P-VALUE

Age, years, mean (SD)	60.1 (12.0)	59.3 (12.6)	61.2 (11.2)	<0.001

Male sex, n (%)	16183 (75.7%)	9482 (79.7%)	6701 (70.7%)	<0.001

History of diabetes, n (%)	9484 (44.4%)			

Current smoking, n (%)	6614 (30.9%)	4531 (38.1%)	2083 (22.0%)	<0.001

History of hypertension, n (%)	10042 (47.0%)	4172 (35.1%)	5870 (61.9%)	<0.001

Peripheral arterial disease, n (%)	211 (1.0%)	70 (0.6%)	141 (1.5%)	<0.001

Transferred from another facility, n (%)	8401 (39.3%)	4778 (40.2%)	3623 (38.2%)	0.003

No insurance, n (%)	15542 (72.7%)	8306 (69.9%)	7236 (76.3%)	<0.001

STEMI, n (%)	13689 (64.0%)	8212 (69.1%)	5477 (57.7%)	<0.001

Symptoms onset-to-arrival, minutes, median (IQR)	246.0 (118.0, 830.5)	225.0 (115.0, 770.0)	290.0 (120.0, 915.0)	<0.001

Weight, mean (SD)	63.4 (9.7)	63.3 (9.8)	63.6 (9.6)	0.039

Systolic BP, mm Hg, mean (SD)	138.5 (29.0)	137.1 (28.1)	140.4 (29.9)	<0.001

Heart rate, per minute, mean (SD)	79.9 (18.9)	78.1 (18.0)	82.3 (19.8)	<0.001

Killip class, n (%)				

I	18459 (86.4%)	10564 (88.9%)	7895 (83.2%)	<0.001

II	1183 (5.5%)	570 (4.8%)	613 (6.5%)	

III	1239 (5.8%)	483 (4.1%)	756 (8.0%)	

IV	492 (2.3%)	272 (2.3%)	220 (2.3%)	

Troponin, ng/mL, median (IQR)	1.3 (0.3, 5.8)	1.4 (0.3, 6.3)	1.2 (0.3, 5.5)	0.021

LDL-C, mg/dL, mean (SD)	122.5 (40.9)	125.3 (39.7)	119.2 (42.2)	<0.001

Triglycerides, mg/dL, median (IQR)	121.0 (90.0, 165.0)	119.0 (88.0, 161.0)	124.0 (90.0, 170.0)	<0.001

Serum Creatinine, mean (SD)	1.2 (0.7)	1.1 (0.5)	1.2 (0.8)	<0.001

Fasting glucose, mg/dL, mean (SD)	148.0 (68.8)	117.6 (41.9)	179.8 (76.6)	<0.001

Hemoglobin, mg/dL, mean (SD)	13.2 (2.0)	13.4 (2.0)	13.0 (2.0)	<0.001

Hospital type, n (%)				

Government	7133 (33.4%)	4582 (38.5%)	2551 (26.9%)	<0.001

Non-profit/Charity	5749 (26.9%)	2920 (24.6%)	2829 (29.8%)	

Private	8492 (39.7%)	4388 (36.9%)	4104 (43.3%)	

Hospital size, n (%)				

Extra Large (>1000 beds)	3560 (16.7%)	2405 (20.2%)	1155 (12.2%)	<0.001

Large (501–1000 beds)	8523 (39.9%)	4456 (37.5%)	4067 (42.9%)	

Medium (201–500 beds)	7415 (34.7%)	4060 (34.1%)	3355 (35.4%)	

Small (≤200 beds)	1876 (8.8%)	969 (8.1%)	907 (9.6%)	

Catherization laboratory, n (%)				

Installed During Study	496 (2.3%)	312 (2.6%)	184 (1.9%)	<0.001

No	3552 (16.6%)	2261 (19.0%)	1291 (13.6%)	

Yes	17326 (81.1%)	9317 (78.4%)	8009 (84.4%)	


Abbreviations: STEMI, ST-segment elevation myocardial infarction; BP, blood pressure; LDL-C, low-density lipoprotein cholesterol.

### Use of evidence-based therapies

Table 2 shows data on the use of evidence-based therapies in patients with and without diabetes. On admission, 97.9% of patients received aspirin and 98.2% received a second antiplatelet agent with no significant difference between those with and without diabetes. Adjuvant anticoagulation therapy was administered to 85.6% of participants but was more likely administered to those with diabetes than those without (84.9% vs. 86.1%; P = 0.012). The utilization of beta-blockers was low in the overall sample (40.1%) and did not differ by diabetes status.

**Table 2:** Frequency of medication use, studies, and procedures during hospitalization and at discharge by diabetes status.


MEASURE OF CARE	TOTAL (n = 21374) n (%)	NO DIABETES (n = 11890) n (%)	DIABETES (n = 9484) n (%)	P-VALUE

**Medications**				

Prehospital aspirin	3796 (17.8%)	1939 (16.3%)	1857 (19.6%)	<0.001

In-hospital aspirin	20885 (97.9%)	11644 (98.1%)	9241 (97.7%)	0.059

In-hospital second antiplatelet	20973 (98.2%)	11673 (98.3%)	9300 (98.2%)	0.74

In-hospital beta-blocker	8314 (40.1%)	4617 (40.0%)	3697 (40.1%)	0.82

In-hospital anticoagulant	18256 (85.6%)	10221 (86.1%)	8035 (84.9%)	0.012

Optimal in-hospital medications *	7000 (33.8%)	3971 (34.5%)	3029 (33.0%)	0.028

**Studies and procedures**				

Echocardiography	19725 (92.3%)	10832 (91.1%)	8893 (93.8%)	<0.001

Diagnostic angiography	12681 (59.3%)	6989 (58.8%)	5692 (60.0%)	0.068

PCI	10553 (49.4%)	6047 (50.9%)	4506 (47.5%)	<0.001

Primary PCI (for STEMI)	6710 (49.0%)	4022 (49.0%)	2688 (49.1%)	0.91

Door-to-balloon time, min, median (IQR) (for STEMI)	83.0 (57.0, 190.0)	75.0 (55.0, 150.0)	91.0 (60.0, 278.0)	<0.001

Thrombolysis (for STEMI)	3167 (23.1%)	2006 (24.4%)	1161 (21.2%)	<0.001

Door-to-needle time, min, median (IQR) (for STEMI)	44.0 (30.0, 70.0)	43.0 (29.0, 66.0)	45.0 (30.0, 80.0)	0.002

Any reperfusion (for STEMI)	9872 (72.1%)	6024 (73.4%)	3848 (70.3%)	<0.001

Rescue PCI (for STEMI)	1675 (12.3%)	1010 (12.3%)	665 (12.2%)	0.78

**Discharge treatments and counseling**				

Discharge aspirin	19137 (97.9%)	10721 (98.0%)	8416 (97.6%)	0.048

Discharge second antiplatelet	19201 (98.0%)	10739 (98.1%)	8462 (97.9%)	0.41

Discharge beta-blocker	12607 (66.1%)	6968 (65.4%)	5639 (66.9%)	0.028

Discharge statin	18989 (97.0%)	10597 (96.8%)	8392 (97.2%)	0.16

Discharge ACE inhibitor or ARB	9232 (48.1%)	5228 (48.5%)	4004 (47.6%)	0.2

Cardiac rehabilitation referral	5684 (28.4%)	3253 (29.1%)	2431 (27.6%)	0.02

Optimal discharge medications **	11937 (63.0%)	6617 (62.5%)	5320 (63.6%)	0.14

Tobacco cessation advice	6144 (95.5%)	4242 (95.7%)	1902 (95.0%)	0.24


Abbreviations: PCI, percutaneous coronary intervention; ACE, angiotensin-converting enzyme; ARB, angiotensin receptor blocker; STEMI, ST-segment elevation myocardial infarction. * Includes the use of aspirin, adjuvant antiplatelet therapy [clopidogrel, prasugrel, or ticagrelor], anticoagulant, and β-blockers; in-hospital statin use was additionally predefined but data were not collected. ** Aspirin, adenosine diphosphate receptor antagonist [clopidogrel, prasugrel, or ticagrelor], statin, and β-blocker.

Among those presenting with STEMI, 72.1% received reperfusion therapy. An equal proportion of STEMI patients with and without diabetes received primary PCI (49.1% vs 49.0%; P = 0.91). However, those with diabetes had significantly longer door-to-needle (median, 45 vs 43 minutes; P = 0.002) and door-to-balloon times (median, 91 vs 75 minutes; P < 0.001). In all comers (STEMI and non-STEMI patients), 12,681 (59.3%) patients had angiography during their hospital stay and 10,553 (49.4%) received PCI. Receiving a PCI during admission was less likely in those with diabetes compared to those without (47.5% vs 50.9%; P < 0.001).

At discharge, most patients (>95%) were prescribed aspirin, an adjuvant antiplatelet agent, statin therapy, and received smoking cessation counseling (for smokers) with no clinically meaningful difference in prescription patterns between those with and without diabetes except for prescribing aspirin, which was lower in patients with diabetes (97.6% vs 98.0; P = 0.048). Those with diabetes were more likely to be discharged on a beta-blocker but less likely to receive a referral to cardiac rehabilitation.

### Adverse in-hospital and 30-day cardiac outcomes

**Figure 1:**
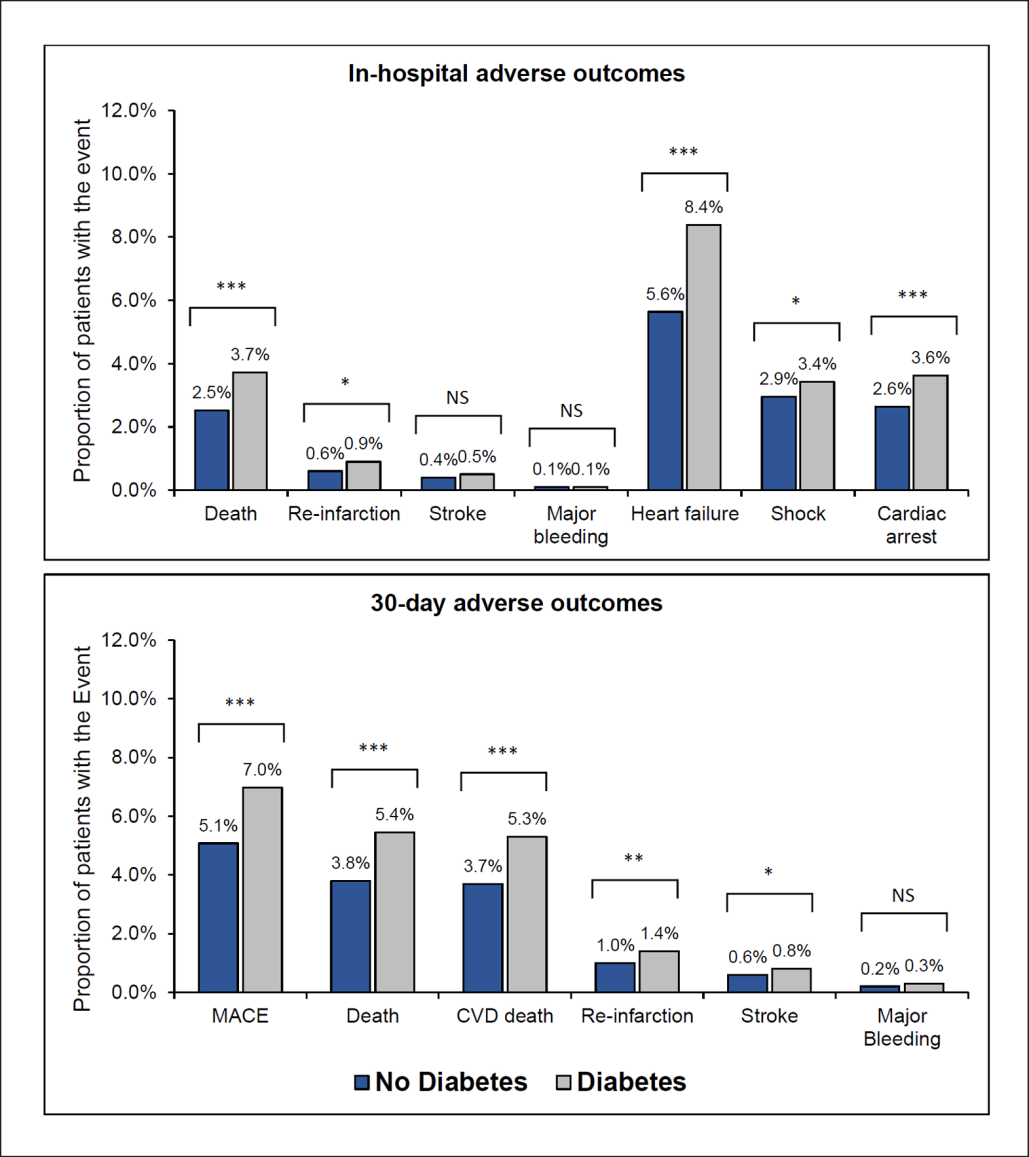
**Frequency of in-hospital and 30-day adverse outcomes by diabetes status**.

The prevalence of in-hospital and 30-day adverse cardiac outcomes in those with and without diabetes is shown in Figure 1 (supplemental Table 2). Among those with and without diabetes, the incidence of heart failure was the most common in-hospital adverse cardiac event (8.4% vs 5.6%; P < 0.001). In those with diabetes, the second most frequent in-hospital adverse cardiac event was death (3.7%) followed by cardiac arrest (3.6%) and cardiogenic shock (3.4%). By contrast, among patients without diabetes, the second most common adverse in-hospital event was cardiogenic shock (3.2%) followed by cardiac arrest (2.6%) and death (2.5%).

At 30-day follow-up, MACE occurred in a total of 1,247 (5.9%) patients and death occurred in a total of 954 (4.5%) patients. CVD death was the most common type of death at 30-days comprising 97.3% of all deaths. MACE, death, CVD death, re-infarction, and stroke were all more prevalent in those with diabetes than those without diabetes. Supplemental Table 3 shows the results stratified by the presence of STEMI in patients with and without diabetes.

Table 3 shows univariable and multivariable adjusted odds ratios for the association between and in-hospital or 30-day adverse events. In an unadjusted model, compared to those without diabetes, individuals with diabetes had a statistically significant 50% higher odds of in-hospital death (OR, 1.5; 05% CI, 1.2 to 1.8), 47% higher odds of in-hospital re-infarction, (OR, 1.47; 95% CI 1.1 to 1.9), 53% higher odds of in-hospital heart failure (OR, 1.5; 95% CI, 1.3 to 1.8), and 39% higher odds for in-hospital cardiac arrest (OR, 1.4; 95% CI, 1.1 to 1.7). These associations remained statistically significant after adjusting for clinical presentation factors and therapies received during admission. Diabetes was not statistically associated with in-hospital stroke, major bleeding, or cardiogenic shock in any of the models.

**Table 3:** Univariable and multivariable models for the association between diabetes and in-hospital and 30-day adverse outcomes.


	MODEL 1		MODEL 2		MODEL 3		MODEL 4	
			
	OR (95% CI)	P-VALUE	OR (95% CI)	P-VALUE	OR (95% CI)	P-VALUE	OR (95% CI)	P-VALUE

**In Hospital outcomes**								

Death	1.50 (1.22 to 1.84)	<0.001	1.40 (1.12 to 1.76)	0.004	1.46 (1.12 to 1.89)	0.004	NA	

Re-infarction	1.47 (1.11 to 1.94)	0.006	1.55 (1.15 to 2.10)	0.004	1.52 (1.15 to 2.02)	0.003	NA	

Stroke	1.08 (0.71 to 1.63)	0.719	1.02 (0.67 to 1.53)	0.938	1.00 (0.66 to 1.51)	0.999	NA	

Major bleeding*	1.00 (0.42 to 2.40)	0.995	0.87 (0.32 to 2.32)	0.776	0.83 (0.31 to 2.23)	0.717	NA	

Heart failure	1.53 (1.28 to 1.83)	<0.001	1.24 (1.04 to 1.46)	0.013	1.26 (1.06 to 1.48)	0.008	NA	

Cardiogenic shock	1.17 (0.91 to 1.50)	0.213	1.18 (0.97 to 1.45)	0.100	1.19 (0.96 to 1.48)	0.105	NA	

Cardiac arrest	1.39 (1.13 to 1.72)	0.002	1.29 (1.02 to 1.64)	0.035	1.30 (1.03 to 1.64)	0.026	NA	

**30-day outcomes**								

MACE**	1.40 (1.22 to 1.62)	<0.001	1.29 (1.12 to 1.48)	<0.001	1.33 (1.14 to 1.55)	<0.001	1.28 (1.02 to 1.61)	0.037

Death	1.46 (1.26 to 1.69)	<0.001	1.34 (1.14 to 1.59)	<0.001	1.40 (1.16 to 1.69)	<0.001	1.32 (0.96 to 1.81)	0.090

CVD death	1.46 (1.26 to 1.68)	<0.001	1.34 (1.14 to 1.58)	0.001	1.40 (1.16 to 1.69)	<0.001	1.28 (0.93 to 1.76)	0.126

Re-infarction	1.41 (1.08 to 1.84)	0.013	1.42 (1.10 to 1.84)	0.007	1.44 (1.13 to 1.85)	0.004	1.33 (0.94 to 1.89)	0.105

Stroke	1.40 (0.95 to 2.05)	0.087	1.31 (0.87 to 1.97)	0.202	1.28 (0.86 to 1.92)	0.222	1.05 (0.53 to 2.11)	0.880

Major bleeding*	1.31 (0.76 to 2.25)	0.335	1.19 (0.66 to 2.12)	0.566	1.17 (0.67 to 2.04)	0.584	0.92 (0.42 to 2.04)	0.842


Model 1: adjusted for clustering by center. Model 2: adjusted for factor in Model 1 plus age, sex, smoking status, presence of STEMI, systolic blood pressure, heart rate, Killip class on presentation. Model 3: adjusted for factors in Model 2 plus administration of aspirin and anticoagulation therapy on admission, PCI during admission, randomization arm. Model 4: adjusted for factors in Model 3 plus discharge aspirin, second antiplatelet, beta-blocker, ACE inhibitor or ARB, statin, and referral to cardiac rehabilitation. Model 4 only included those who survived their hospitalization. * Major bleeding defined as defined by the Global Utilization of Streptokinase and Tissue Plasminogen Activator for Occluded Coronary Arteries [GUSTO] criteria,16 which is defined by intracerebral hemorrhage or bleeding resulting in substantial hemodynamic compromise requiring treatment. ** Defined as death, reinfarction (defined by the Third Universal Definition of Myocardial Infarction13), stroke, and major bleeding (defined by the Global Utilization of Streptokinase and Tissue Plasminogen Activator for Occluded Coronary Arteries [GUSTO] criteria,16 which is defined by intracerebral hemorrhage or bleeding resulting in substantial hemodynamic compromise requiring treatment).

Those with diabetes had a statistically significant 40% higher odds of 30-day MACE (OR, 1.4; 95% CI, 1.2 to 1.6), 46% higher odds of 30-day death (OR, 1.5; 95% CI, 1.3 to 1.7), 46% higher odds of 30-day CVD death (OR, 1.5; 95% CI, 1.3 to 1.7) and 41% higher odds of 30-day re-infarction (OR, 1.4; 95% CI, 1.1 to 1.8). These associations remained statistically significant after adjusting for baseline clinical factors (Model 2) and important therapies received during admission (Model 3). Moreover, diabetes was significantly associated with higher 30-day MACE after additional adjustment for therapies on discharge (Model 4) (adjusted OR, 1.28; 95% CI, 1.0 to 1.6).

**Figure 2:**
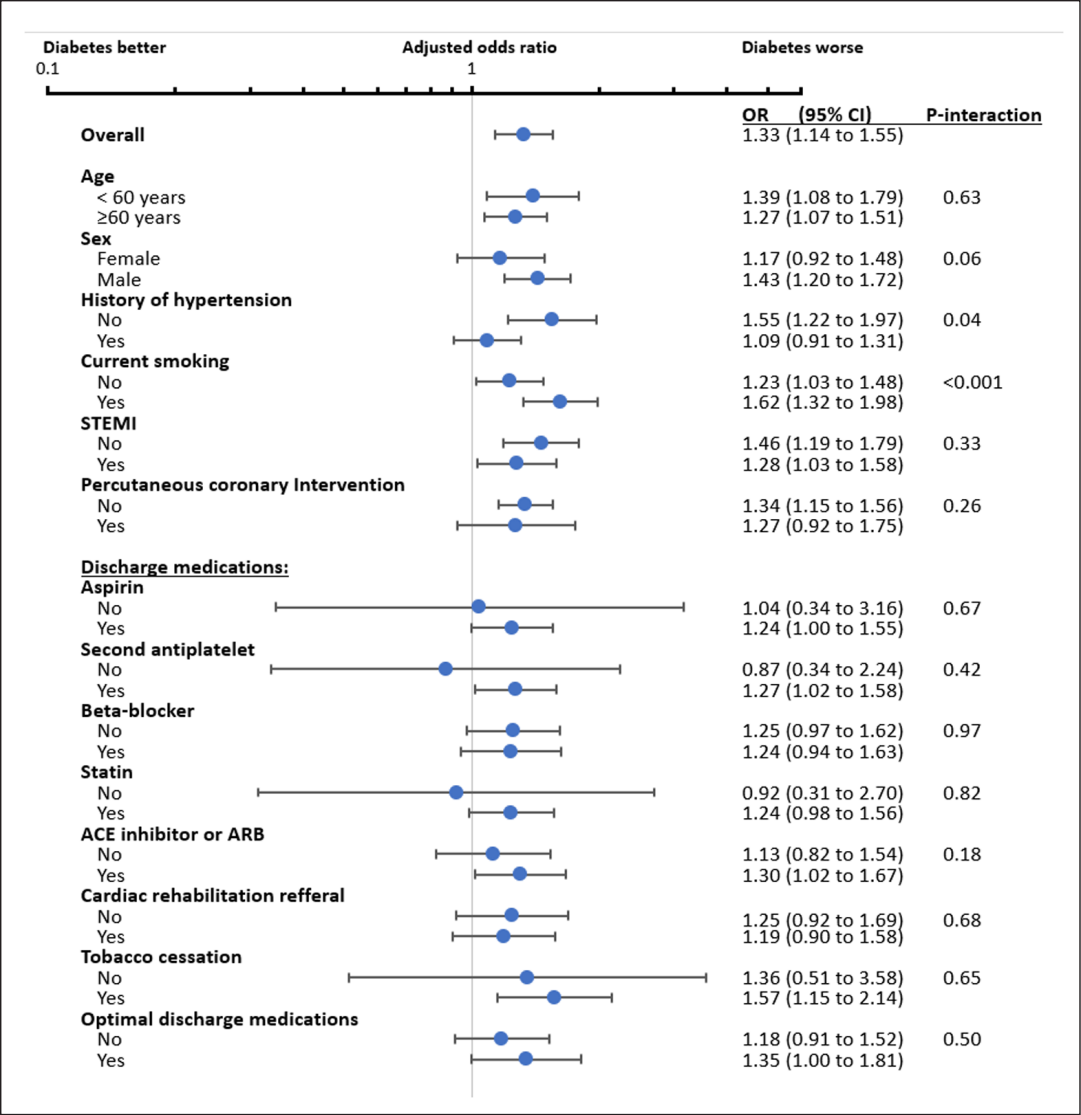
**Multivariable adjusted odds ratios for the association between diabetes and 30-day MACE after myocardial infarction in selected subgroups**. Abbreviations: PCI, percutaneous coronary intervention; STEMI, ST-segment elevation myocardial infarction. Adjusted OR were calculated using a cluster-adjusted multivariable logistic model which included age, sex, smoking status, presence of STEMI, systolic blood pressure, heart rate, Killip class on presentation, administration of aspirin, and anticoagulation therapy on admission, PCI during admission, randomization arm.

Figure 2 shows the multivariable adjusted odds ratios for 30-day MACE and diabetes stratified by demographic and treatment factors. Two significant interactions were noted. First, diabetes was associated with higher 30-day MACE in those without hypertension but not in those with hypertension (P-interaction = 0.04). Second, the association between diabetes and higher MACE was stronger for those who were current smokers than for those who were not current smokers (P-interaction < 0.001). Supplemental Table 4 and supplementary Table 5 show the number of events according to diabetes status and stratified by history of hypertension and current smoking, respectively. Supplemental Figure 1 shows the multivariable adjusted probability of 30-day MACE across age in patients with and without diabetes stratified by history of hypertension and current smoking.

## Discussion

In this analysis, we examined the impact of diabetes on CV outcomes after AMI using data from the ACS QUIK trial in Kerala India. In patients presenting with myocardial infarction, diabetes was highly prevalent and associated with a significant increase in 30-day MACE. This finding was independent of clinical characteristics on presentation, in-hospital therapies received, and discharge medications. Although the association between diabetes and 30-day MACE was consistent when examined in important patient subgroups, the association was stronger in current smokers than in non-smokers, and present in those without hypertension but not in those with hypertension. Diabetes was also significantly and independently associated with higher in-hospital morbidity and mortality including higher likelihood of in-hospital reinfarction, heart failure, and cardiac arrest after myocardial infarction.

Recent disease trends in LMICs show a significant rise in both diabetes and CVD [2]. In India alone, the prevalence of diabetes has doubled in the past 20 years and the number of those with diabetes is estimated to reach 65.1 million in 2030 [12]. CVD, a well-known complication of diabetes, is now the leading cause of death in India, with CVD death rates that exceed those seen in western countries [13]. Therefore, understanding the impact of diabetes on CVD outcomes is of considerable public health importance.

In the current analysis, we observed a higher prevalence of diabetes (44%) in myocardial infarction patients than in the Kerala ACS Registry (36.1% with diabetes) which collected data from form the same region in India between May 2007 to May 2009 [14]. This difference highlights a potential rise in the prevalence of diabetes in the decade between the two studies. Prior registries from Western countries and China show a lower percentage of diabetes among ACS patients but registries from the Middle East show comparable percentages [15,16,17].

### Differences in presentation and initial treatments

Consistent with prior studies, we show that patients with diabetes were more likely to be older and female [16]. Prior studies have shown that these factors together with diabetes are associated with more frequent atypical ischemic symptoms, which may explain why patients with diabetes in our study had significant delays in hospital presentation [18]. We also observe that patients with diabetes have a higher Killip class despite lower proportions of STEMI [17]. This has been observed in western ACS registries and may be due to the higher burden of ischemia in patients with diabetes or delayed hospital presentation in the face of ongoing myocardial necrosis due to myocardial infarction.

In patients with and without diabetes, the morbidity and mortality associated with STEMI is significantly reduced with prompt reperfusion [19]. Current guidelines advocate for achieving door-to-balloon times ≤90 minutes and, for non-PCI capable hospitals, a door-to-needle times ≤30 minutes when the anticipated time to mechanical reperfusion is >120 minutes. In our study, STEMI patients with and without diabetes were equally likely to receive primary PCI. However, STEMI patients with diabetes had significantly longer door-to-balloon times with more than half of those with diabetes having door-to-balloon times >90 min or door-to-needle times >45 min. These delays occurring after hospital presentation compound earlier delays from symptom onset-to-hospital presentation. Other studies have reported similar delays in reperfusion in those with diabetes [20,21].

Among the various factors delaying reperfusion, the struggle to afford therapy costs is perhaps a critical issue in Kerala. In a substudy of ACS-QUIK addressing the microeconomics of AMI, 54% of patients experienced catastrophic health spending that exceeded 40% of the patient’s annual household expenditure, excluding food costs; 8% had to either sell or mortgage assets or borrow money to buffer the cost of AMI treatment [22]. As expected, these findings were more pronounced in those without baseline health insurance. In our study, those with diabetes were less likely to have health insurance and, therefore, more likely to face greater financial challenges, perhaps contributing to delays in reperfusion. Door-to-reperfusion (whether mechanical or pharmacologic) is an actionable measure of cardiovascular care. Our findings should catalyze institutional quality improvement aiming at achieving timely reperfusion in patients with diabetes. Enhancing access to healthcare insurance to facilitate the timely delivery of AMI therapies should be considered, especially among higher-risk populations such as those with diabetes.

### Differences in outcomes between those with and without diabetes

The current study confirms the significant morbidity and mortality associated with diabetes reported in prior studies and extends these findings to patients in LMIC [20,23,24]. Our findings show that diabetes is independently associated with higher adverse outcomes and contradicts the findings of other studies suggesting diabetes was not associated with increased risk after adjusting for the lower utilization of evidence-based treatments and PCI in patients with diabetes [25,26]. Diabetes has been associated with higher coronary artery disease burden and extreme serum glucose values at the time of AMI, which were associated with worse prognosis [26]. Diabetes is also associated with a higher incidence of heart failure, and cardiac arrest after AMI [27,28,29]. These associations were confirmed in the current study. Our findings highlight the importance of emphasizing primary and secondary CVD prevention in patients with diabetes. Future studies should examine the efficacy of different strategies aiming at enhancing primary and secondary prevention among patients with diabetes in LMIC.

An interesting observation of the current analysis was the significant interaction between diabetes and a history of hypertension or smoking with regards to 30-day MACE. In those without hypertension, diabetes was associated with higher 30-day MACE. However, in those with hypertension, events were not different in those with and without diabetes. Both hypertension and diabetes are strong CVD risk factors and are associated with a pro-thrombotic state and impaired left ventricular function [30,31,32]. Data from observational studies suggest a synergistic interaction between hypertension and diabetes with regards to stroke [33], kidney function worsening, and heart failure [34]. By contrast, a pooled analysis of four randomized clinical trials including 28,771 patients with MI and evidence of heart failure or left ventricular dysfunction found no significant interaction between history of diabetes and hypertension with regards to adverse cardiac outcomes [35]. Our finding of a sub-additive interaction between hypertension and diabetes was unexpected. Both hypertension and diabetes may be manifestations of an underlying adverse cardiometabolic state that is prevalent in the study population. As a result, our findings may be due to collinearity among these variables contributing to the risk of adverse events.

The presence of both diabetes and current smoking was adversely synergistic on 30-day MACE. Data from Nurses’ Health Study suggested that the association between diabetes and CHD was weaker in female smokers than non-smokers, while other studies including both males and females have shown no interaction [36,37,38]. Our finding is important, especially that tobacco use is growing the fastest in LMICs and was present in 22% of those with diabetes in our sample [39]. This interaction highlights the importance of counseling patients with diabetes on tobacco cessation. Reassuringly, in our study, receiving tobacco cessation advice on discharge was equally high (>95%) in those with and without diabetes. However, the percentage of those achieving tobacco cessation at 30-day follow-up is unclear. Future studies should investigate the effectiveness of implementing post-MI smoking cessation interventions in low- or middle-income countries.

### Non-STEMI patients with diabetes are at high risk of cardiac events

Among non-STEMI patients, we observed higher in-hospital and 30-day adverse outcomes in those with diabetes compared to those without diabetes. These findings are consistent with prior studies of patients with non-STEMI [16,40,41]. Furthermore, non-STEMI patients with diabetes have as high in-hospital and 30-day adverse outcomes as STEMI patients without diabetes. In the acute phase of non-STEMI management, the current AHA/ACC guideline recommends that clinical decisions for medical therapy and revascularization should be similar in those with and without diabetes [42].

Clinical risk scores to risk stratify non-STEMI patients such as the TIMI risk score or GRACE risk calculator patients do not account for diabetes status [43,44]. Our findings that diabetes was independently associated with the risk of adverse outcomes suggests the importance of accounting for diabetes status at the time of risk stratification. Moreover, our findings that the proportion of in-hospital heart failure was the highest in non-STEMI patients with diabetes highlights the significant morbidity associated with diabetes that is not captured by risk scores.

### Strengths and Limitations

A major strength of our study is that it leverages data from the largest trial in India to date. Furthermore, the high prevalence of diabetes, smoking, and hypertension allow for exploring their interaction with regards to 30-day MACE. Our study has some notable limitations. This was a post-hoc analysis of data from ACS QUIK. Although we used multivariable regression to adjust for potential confounding, our analysis is outside of randomization making unmeasured or residual confounding possible. Second, we analyzed the difference in tobacco cessation advice and cardiac rehabilitation referral between those with and without diabetes. However, data on the percent of patients who were successful in achieving tobacco cessation or receiving cardiac rehabilitation are missing; thus, the association between tobacco cessation or cardiac rehabilitation and 30-day MACE cannot be assessed. Cardiac rehabilitation in India remains grossly underutilized and the number of cardiac rehabilitation centers remains insufficient; as such, efforts to improve the uptake of cardiac rehabilitation in India are needed [45]. Furthermore, we have no data on adherence to discharge medication or the patient’s ability to schedule post-discharge follow-up, both important predictors of lower adverse events after myocardial infarction.

Third, while we report on the percentage of patients receiving PCI, data on the angiographic difference between those with and without diabetes were not collected. Lastly, although the ACS QUIK was a cluster-randomized clinical trial at the hospital center level, informed consent was required for 30-day follow-up. This might have resulted in some recruitment bias. However, Huffman et al. showed that baseline characteristics were balanced in those with and without 30-day follow, except for troponin values which were higher in those missing 30-day follow-up (median, 1.3 vs 4.6 ng/ml) [46].

## Conclusion

Among patients presenting with AMI in India, a considerable proportion has diabetes and are at increased risk for in-hospital and 30-day adverse CV outcomes. The attainment of guideline-driven therapeutic goals such as prompt reperfusion may not be achieved in patients with diabetes and AMI who may more often present with atypical symptoms and HF. Increased awareness of the increased CV risk and attention to the implementation of established CV therapies are indicated for patients with diabetes in LMICs who develop an ACS.

## Additional File

The additional file for this article can be found as follows:

10.5334/gh.1290.s1 Supplementary material. Supplemental Tables 1to 5 and Supplemental Figure 1.
