# Supplementary material for: Cardiovascular Outcomes Among Patients with Acute Coronary Syndromes and Diabetes: Results from ACS QUIK Trial in India
Source: Glob Heart. 2024 Apr 24;19(1):37. doi: 10.5334/gh.1290 (PMC11049669; doi:10.5334/gh.1290)
Supplement: Supplementary material. — Supplemental Tables 1to 5 and Supplemental Figure 1. [file gh-19-1-1290-s1.pdf]

Supplemental Table 1. Baseline demographic and clinical characteristics in patients with STEMI by diabetes status

	STEMI patient without diabetes (n=8212)	STEMI patient with diabetes (n=5477)	P-value
Age, years, mean (SD)	58.2 (12.4)	59.5 (10.8)	<0.001
Male sex, n (%)	6764 (82.4%)	4079 (74.5%)	<0.001
Current smoking, n (%)	3420 (41.6%)	1373 (25.1%)	<0.001
Symptom onset-to-arrival, minutes, median (IQR)	180.0 (105.0, 460.0)	210.0 (108.0, 661.0)	<0.001
Weight, mean (SD)	63.9 (9.5)	64.0 (9.3)	0.44
Systolic BP, mm Hg, mean (SD)	134.8 (27.1)	136.5 (29.0)	<0.001
Heart rate, per minute, mean (SD)	76.8 (16.8)	80.0 (18.9)	<0.001
Killip class, n (%)			<0.001
I	7312 (89.0%)	4677 (85.4%)	
II	431 (5.2%)	385 (7.0%)	
III	220 (2.7%)	233 (4.3%)	
IV	249 (3.0%)	182 (3.3%)	
Troponin, ng/mL, median (IQR)	2.1 (0.4, 10.0)	2.3 (0.4, 10.0)	0.52
LDL-C, mg/dL, mean (SD)	127.0 (38.9)	123.0 (40.9)	<0.001
Triglycerides, mg/dL , median (IQR)	118.0 (88.0, 161.0)	124.0 (90.0, 170.0)	<0.001
Serum Creatinine, mean (SD)	1.1 (0.4)	1.1 (0.6)	<0.001
Fasting glucose, mg/dL , mean (SD)	119.1 (42.0)	184.2 (76.6)	<0.001
Hemoglobin, mg/dL, mean (SD)	13.5 (1.9)	13.3 (2.0)	<0.001

Abbreviations: STEMI, ST-segment elevation myocardial infarction; BP, blood pressure; LDL-C, low-density lipoprotein cholesterol.

Supplemental Table 2. In-hospital and 30-day adverse cardiac events by diabetes status.

Outcome	Total (n=21374) n (%)	No diabetes (n=11890) n (%)	Diabetes (n=9484) n (%)	P-value
**In Hospital outcomes**				
Death	652 (3.1%)	299 (2.5%)	353 (3.7%)	<0.001
Re-infarction	167 (0.8%)	77 (0.6%)	90 (0.9%)	0.013
Stroke	93 (0.4%)	50 (0.4%)	43 (0.5%)	0.72
Major bleeding*	27 (0.1%)	15 (0.1%)	12 (0.1%)	0.99
Heart failure	1465 (6.9%)	670 (5.6%)	795 (8.4%)	<0.001
Cardiogenic shock	675 (3.2%)	350 (2.9%)	325 (3.4%)	0.045
Cardiac arrest	657 (3.1%)	313 (2.6%)	344 (3.6%)	<0.001
**30-day outcomes**				
MACE **	1247 (5.9%)	595 (5.1%)	652 (7.0%)	<0.001
Death	954 (4.5%)	445 (3.8%)	509 (5.4%)	<0.001
CVD death	928 (4.4%)	433 (3.7%)	495 (5.3%)	<0.001
Re-infarction	256 (1.2%)	121 (1.0%)	135 (1.4%)	0.007
Stroke	150 (0.7%)	71 (0.6%)	79 (0.8%)	0.04
Major bleeding *	49 (0.2%)	24 (0.2%)	25 (0.3%)	0.35

* Major bleeding defined as defined by the Global Utilization of Streptokinase and Tissue Plasminogen Activator for Occluded Coronary Arteries [GUSTO] criteria,16 which is defined by intracerebral hemorrhage or bleeding resulting in substantial hemodynamic compromise requiring treatment.

** defined as death, reinfarction (defined by the Third Universal Definition of Myocardial Infarction13), stroke, and major bleeding (defined by the Global Utilization of Streptokinase and Tissue Plasminogen Activator for Occluded Coronary Arteries [GUSTO] criteria,16 which is defined by intracerebral hemorrhage or bleeding resulting in substantial hemodynamic compromise requiring treatment)

Supplemental Table 3. In-hospital and 30-day adverse cardiac events by STEMI and diabetes status.

	Non-STEMI			STEMI		
	**No diabetes (n=3678)**	**Diabetes (n=4007)**	**P-value**	**No diabetes (n=8212)**	**Diabetes (n=5477)**	**P-value**
**In Hospital outcomes**						
In-hospital Death	46 (1.3%)	100 (2.5%)	<0.001	253 (3.1%)	253 (4.6%)	<0.001
Re-infarction	12 (0.3%)	27 (0.7%)	0.032	65 (0.8%)	63 (1.2%)	0.033
Stroke	14 (0.4%)	22 (0.5%)	0.28	36 (0.4%)	21 (0.4%)	0.62
Major bleeding***	3 (0.1%)	5 (0.1%)	0.56	12 (0.1%)	7 (0.1%)	0.78
Heart failure	207 (5.6%)	347 (8.7%)	<0.001	463 (5.6%)	448 (8.2%)	<0.001
Cardiogenic shock	34 (0.9%)	61 (1.5%)	0.018	316 (3.8%)	264 (4.8%)	0.006
Cardiac arrest	40 (1.1%)	93 (2.3%)	<0.001	273 (3.3%)	251 (4.6%)	<0.001
**30-day outcomes**						
MACE **	141 (3.9%)	241 (6.1%)	<0.001	454 (5.6%)	411 (7.6%)	<0.001
Death	101 (2.8%)	179 (4.5%)	<0.001	344 (4.2%)	330 (6.1%)	<0.001
CVD death	95 (2.6%)	172 (4.4%)	<0.001	338 (4.2%)	323 (6.0%)	<0.001
Re-infarction	30 (0.8%)	50 (1.3%)	0.063	91 (1.1%)	85 (1.6%)	0.023
Stroke	17 (0.5%)	34 (0.9%)	0.038	54 (0.7%)	45 (0.8%)	0.26
Major bleeding*	4 (0.1%)	9 (0.2%)	0.22	20 (0.2%)	16 (0.3%)	0.58

Abbreviation: STEMI, ST-segment elevation myocardial infarction; MACE, major adverse cardiac events; CVD, cardiovascular disease.

* Major bleeding defined as defined by the Global Utilization of Streptokinase and Tissue Plasminogen Activator for Occluded Coronary Arteries [GUSTO] criteria,16 which is defined by intracerebral hemorrhage or bleeding resulting in substantial hemodynamic compromise requiring treatment.

** defined as death, reinfarction (defined by the Third Universal Definition of Myocardial Infarction13), stroke, and major bleeding (defined by the Global Utilization of Streptokinase and Tissue Plasminogen Activator for Occluded Coronary Arteries [GUSTO] criteria,16 which is defined by intracerebral hemorrhage or bleeding resulting in substantial hemodynamic compromise requiring treatment)

Supplemental Table 4. In-hospital and 30-day adverse cardiac events by history of hypertension and diabetes status.

	No hypertension			Hypertension		
	**No diabetes (n=7718) n (%)**	**Diabetes (n=3614) n (%)**	**P-value**	**No diabetes (n=4172) n (%)**	**Diabetes (n=5870) n (%)**	**P-value**
**In Hospital outcomes**						
Death	180 (2.3%)	138 (3.8%)	<0.001	119 (2.9%)	215 (3.7%)	0.026
Re-infarction	48 (0.6%)	33 (0.9%)	0.086	29 (0.7%)	57 (1.0%)	0.14
Stroke	22 (0.3%)	14 (0.4%)	0.37	28 (0.7%)	29 (0.5%)	0.24
Major bleeding*	7 (0.1%)	3 (0.1%)	0.9	8 (0.2%)	9 (0.2%)	0.64
Heart failure	382 (4.9%)	278 (7.7%)	<0.001	288 (6.9%)	517 (8.8%)	<0.001
Cardiogenic shock	228 (3.0%)	137 (3.8%)	0.019	122 (2.9%)	188 (3.2%)	0.43
Cardiac arrest	181 (2.3%)	134 (3.7%)	<0.001	132 (3.2%)	210 (3.6%)	0.26
**30-day outcomes**						
MACE **	340 (4.5%)	248 (7.0%)	<0.001	255 (6.2%)	404 (7.0%)	0.12
Death	262 (3.4%)	195 (5.5%)	<0.001	183 (4.4%)	314 (5.4%)	0.027
CVD death	255 (3.4%)	192 (5.4%)	<0.001	178 (4.3%)	303 (5.2%)	0.037
Re-infarction	68 (0.9%)	50 (1.4%)	0.014	53 (1.3%)	85 (1.5%)	0.45
Stroke	34 (0.4%)	30 (0.8%)	0.01	37 (0.9%)	49 (0.8%)	0.78
Major bleeding *	12 (0.2%)	8 (0.2%)	0.44	12 (0.3%)	17 (0.3%)	0.98

* Major bleeding defined as defined by the Global Utilization of Streptokinase and Tissue Plasminogen Activator for Occluded Coronary Arteries [GUSTO] criteria,16 which is defined by intracerebral hemorrhage or bleeding resulting in substantial hemodynamic compromise requiring treatment.

** defined as death, reinfarction (defined by the Third Universal Definition of Myocardial Infarction13), stroke, and major bleeding (defined by the Global Utilization of Streptokinase and Tissue Plasminogen Activator for Occluded Coronary Arteries [GUSTO] criteria,16 which is defined by intracerebral hemorrhage or bleeding resulting in substantial hemodynamic compromise requiring treatment).

Supplemental Table 5. In-hospital and 30-day adverse cardiac events by current smoking and diabetes status.

	Not a current smoker			Current smoker		
	**No diabetes (n=7359) n (%)**	**Diabetes (n=7401) n (%)**	**P-value**	**No diabetes (n=4531) n (%)**	**Diabetes (n=2083) n (%)**	**P-value**
**In Hospital outcomes**						
Death	215 (2.9%)	279 (3.8%)	0.004	84 (1.9%)	74 (3.6%)	<0.001
Re-infarction	45 (0.6%)	55 (0.7%)	0.33	32 (0.7%)	35 (1.7%)	<0.001
Stroke	33 (0.4%)	29 (0.4%)	0.6	17 (0.4%)	14 (0.7%)	0.1
Major bleeding*	13 (0.2%)	8 (0.1%)	0.27	2 (<1%)	4 (0.2%)	0.063
Heart failure	443 (6.0%)	629 (8.5%)	<0.001	227 (5.0%)	166 (8.0%)	<0.001
Cardiogenic shock	201 (2.7%)	231 (3.1%)	0.16	149 (3.3%)	94 (4.5%)	0.014
Cardiac arrest	220 (3.0%)	272 (3.7%)	0.02	93 (2.1%)	72 (3.5%)	<0.001
**30-day outcomes**						
MACE **	415 (5.7%)	504 (6.9%)	0.003	180 (4.0%)	148 (7.2%)	<0.001
Death	319 (4.4%)	393 (5.4%)	0.005	126 (2.8%)	116 (5.7%)	<0.001
CVD death	312 (4.3%)	385 (5.3%)	0.005	121 (2.7%)	110 (5.4%)	<0.001
Re-infarction	76 (1.0%)	89 (1.2%)	0.32	45 (1.0%)	46 (2.3%)	<0.001
Stroke	47 (0.6%)	61 (0.8%)	0.18	24 (0.5%)	18 (0.9%)	0.11
Major bleeding *	19 (0.3%)	19 (0.3%)	0.99	5 (0.1%)	6 (0.3%)	0.099

* Major bleeding defined as defined by the Global Utilization of Streptokinase and Tissue Plasminogen Activator for Occluded Coronary Arteries [GUSTO] criteria,16 which is defined by intracerebral hemorrhage or bleeding resulting in substantial hemodynamic compromise requiring treatment.

** defined as death, reinfarction (defined by the Third Universal Definition of Myocardial Infarction13), stroke, and major bleeding (defined by the Global Utilization of Streptokinase and Tissue Plasminogen Activator for Occluded Coronary Arteries [GUSTO] criteria,16 which is defined by intracerebral hemorrhage or bleeding resulting in substantial hemodynamic compromise requiring treatment.

Supplemental Figure 1. Multivariable adjusted probability of 30-day MACE after myocardial infarction in patients with and without diabetes across age stratified by history of hypertension and current smoking.

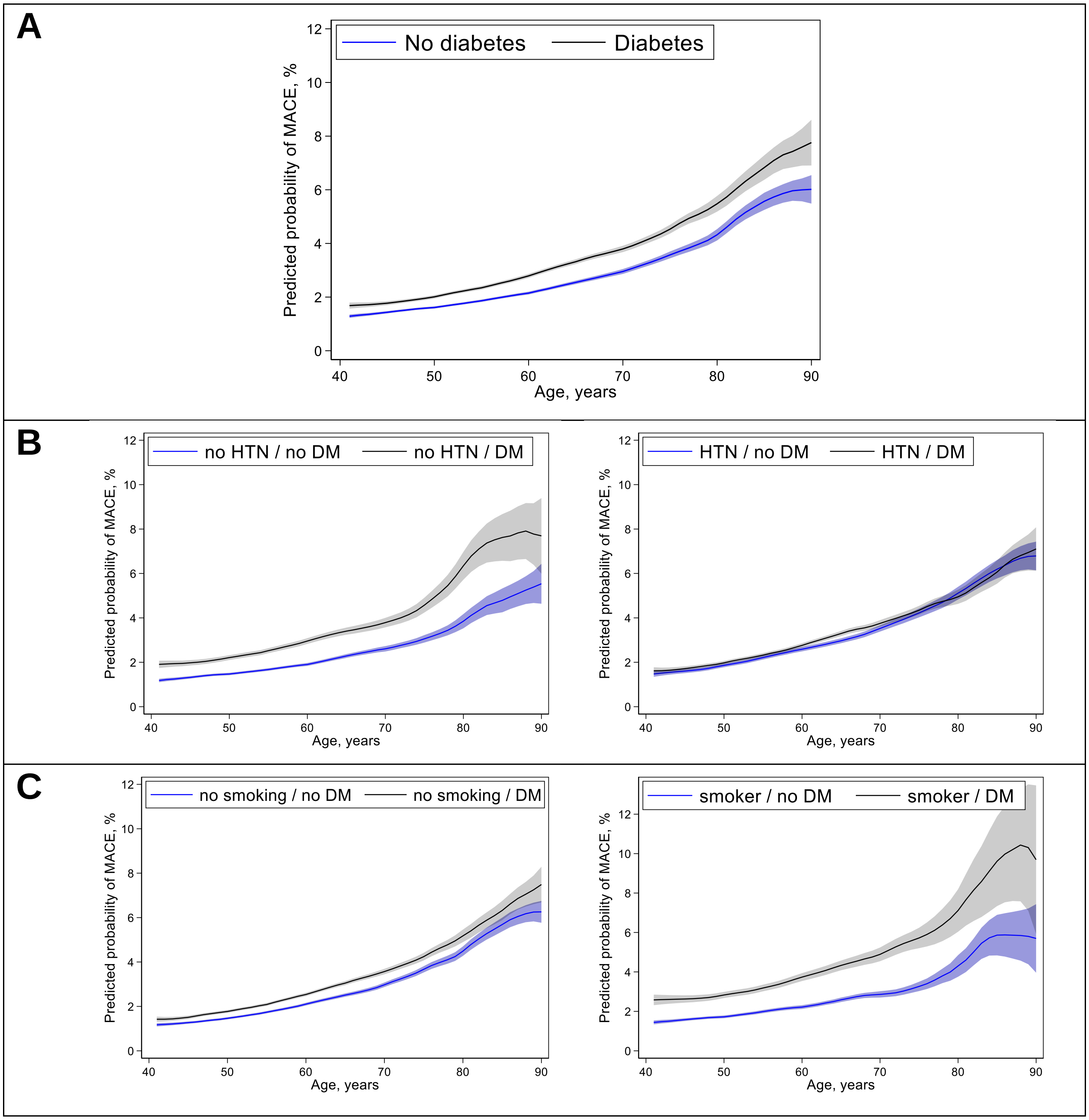

Panel A shows the average (line) and 95% CI (shaded area) of the predicted probability of 30-day MACE in those with and without diabetes. Panel B is stratified by history of hypertension (diabetes-hypertension $P_{interaction}$ = 0.04), and panel C is stratified by current smoking (diabetes-smoking $P_{interaction}$<0.001). Probabilities were calculated using cluster-adjusted multivariable logistic regression which the factors included in Model 4 plus an interaction term between the stratifying factor and age
